# Cryptococcosis in Patients following Kidney Transplantation: A 9-Year Retrospective Clinical Analysis at a Chinese Tertiary Hospital

**DOI:** 10.1155/2019/7165160

**Published:** 2019-11-11

**Authors:** Meifang Yang, Xuan Zhang, Jianhua Hu, Hong Zhao, Lanjuan Li

**Affiliations:** ^1^State Key Laboratory for Diagnosis and Treatment of Infectious Diseases, The First Affiliated Hospital, School of Medicine, Zhejiang University, Hangzhou, China; ^2^Collaborative Innovation Center for Diagnosis and Treatment of Infectious Diseases, No. 79 Qingchun Road, Hangzhou 310003, China

## Abstract

**Background:**

Cryptococcosis following kidney transplantation (KT) is rare but is associated with considerably increased risk of mortality. At present, data on the association between cryptococcosis and KT in mainland China remain relatively limited.

**Objectives:**

This study aims to review our experience related to the management of cryptococcosis following KT at a Chinese tertiary hospital.

**Methods:**

All patients with cryptococcosis following KT admitted to our hospital from January 2010 to December 2018 were reviewed.

**Results:**

A total of 37 patients with cryptococcosis were enrolled (males: 62.2%). The mean age of the patients was 49.5 ± 9.38 (20–64) years. The average time to infection following KT was 7.0 ± 5.50 years (5 months to 21 years), and 30 patients (81.1%) had cryptococcosis onset >2 years following transplantation. The most common site of infection was the central nervous system, followed by the pulmonary system and skin. Most patients received fluconazole or voriconazole with or without flucytosine as their initial treatment regimen at our hospital. The 2-week mortality rate was 8.1% (3/37), and five patients (13.5%) died within 6 months of being diagnosed with cryptococcosis. Remarkably, all patients who received high-dose fluconazole (800 mg daily) or voriconazole ± flucytosine survived.

**Conclusions:**

Cryptococcosis in kidney transplant recipients is typically a late-occurring infection, with most patients having cryptococcosis onset >2 years following KT at our hospital. The central nervous system, pulmonary system, and skin are the main sites of infection. Voriconazole or high-dose fluconazole can be used as an alternative therapy for post-KT cryptococcosis.

## 1. Introduction

Cryptococcosis is an important opportunistic fungal infection responsible for significant mortality and morbidity among solid organ transplant (SOT) recipients, including kidney transplant recipients [1–3]. Several studies have demonstrated that cryptococcosis ranks the third most common cause of invasive fungal infection among SOT recipients [1, 4, 5]. With the improvements in transplantation practices and the wider use of antifungal prophylaxis, the incidence rates of invasive candidiasis and aspergillosis have substantially decreased in SOT recipients in the past decade [4, 6]. However, there has been no change in the incidence of cryptococcosis in SOT recipients [4]. China is one of the countries which perform a high number of kidney transplantations (KTs) worldwide; however, invasive fungal infection remains an important cause of death of KT recipients [7]. According to a previous review from China [8], the incidence of cryptococcosis in KT recipients was estimated to be 0.76% in China. However, few cases of cryptococcosis in patients who have undergone KT have been reported in China [9–11]. Therefore, the characteristics of cryptococcosis in KT recipients in China are not well defined.

Here, we retrospectively reviewed a series of patients with post-KT cryptococcosis admitted to our hospital from 2010 to 2018 to evaluate the clinical characteristics, laboratory findings, diagnoses, treatments, and outcomes of cryptococcosis in this population.

## 2. Patients and Methods

### 2.1. Case Collection

This retrospective study was conducted at the First Affiliated Hospital of Zhejiang University, a 2,500-bed tertiary hospital located in Hangzhou, China, which is considered a top KT centre and centre for infectious diseases in China. We perform approximately 350–400 KTs per year. The medical records of all patients with cryptococcal infection following KT admitted to our hospital from January 1, 2010, to December 1, 2018, were collected. We reviewed patient records including demographic data, underlying diseases, clinical manifestations, laboratory findings, image findings, antifungal treatments, and outcomes.

Cases of cryptococcosis were defined as those in whom the identification of positive findings on *Cryptococcus* culture was obtained from any site or India ink staining in cerebrospinal fluid (CSF), histopathological findings showing the presence of 5–10 *μ*m encapsulated yeasts within tissues or a positive result on cryptococcal antigen testing. Disseminated cryptococcosis is defined as the involvement of at least two organ systems or the presence of fungemia.

The present study was approved by the Ethics Committee of the First Affiliated Hospital of Zhejiang University and was performed in compliance with the Declaration of Helsinki.

### 2.2. Treatment and Outcomes

Detailed antifungal treatment information, including the details of induction and maintenance therapy, was available. Antifungal therapy was initiated upon the confirmation of diagnosis of cryptococcosis. The induction treatment included liposomal amphotericin B (liposomal-AMB) infusion, fluconazole (FLZ), and voriconazole (VRZ) or itraconazole alone or combined with 5-fluorocytosine (5-FC) for at least 14 days, followed by FLZ or VRZ as maintenance therapy for at least 6 months. The antifungal treatment regime was formulated by the attending physician and was mainly based on the Infectious Diseases Society of America (IDSA) guidelines from 2010 [12]. For the treatment group, we also considered the drug availability, disease severity, organ involvement, liver function, and renal function. The treatment duration was individualized according to a patient's response and the decision of the attending physician.

Following discharge, all patients were followed up until death or for at least 6 months following the administration of the initial antifungal therapy. For patients who died during the follow-up, the date and cause of death were recorded. All-cause mortality at 2 weeks and 6 months following the initiation of antifungal therapy was analyzed.

### 2.3. Statistical Analysis

Continuous variables, including age and time from transplantation to cryptococcosis onset, were presented as mean ± standard deviation. Categorical variables, including sex, were presented as counts and proportions. *T* test, chi-squared test, Fisher's exact test, and Mann–Whitney *U* test were used to compare study variables between groups. All tests of statistical hypotheses were two-sided, and *p* ≤ 0.05 was considered statistically significant. All data were analyzed using SPSS 16.0 (SPSS Inc., Chicago, IL, USA).

## 3. Results

### 3.1. Demographic and Clinical Characteristics

A total of 37 patients with post-KT cryptococcosis were enrolled during the 9-year study period. The demographic characteristics of all patients are summarized in Table 1. The average age at post-KT cryptococcosis onset was 49.5 ± 9.38 (20–64) years. Of these, 23 patients were male (62.2%), and the average time from transplantation to cryptococcosis onset was 7.0 ± 5.50 years (5 months to 21 years). Among the 37 patients, 30 (81.1%) had cryptococcosis onset >2 years following KT.

The most common underlying diseases in the study patients were hypertension (15/37, 40.5%), chronic viral hepatitis B (10/37, 27.0%; 1 patient had decompensated cirrhosis), type 2 diabetes (5/37, 13.5%), chronic viral hepatitis C (3/37, 8.1%), and systemic lupus erythematosus (1/37, 2.7%). None of the patients were positive for HIV.

The immunosuppressive regimens used are listed in Table 1. Three patients received intravenous methylprednisolone (500 mg d1, 320 mg d2, and 240 mg d3) for 3 days because of acute rejection-based biopsy within 2 years before cryptococcosis diagnosis. Graft loss prior to antifungal treatment occurred in 3 patients (8.1%). The average serum creatinine level prior to antifungal treatment was 202.90 ± 153.40 (55–693) *μ*mol·L^−1^, and the baseline creatinine level was >176.8 *μ*mol·L^−1^ in 14 patients (37.8%). Furthermore, the baseline creatinine level of the patients with disseminated cryptococcosis was significantly higher than that of the patients without disseminated cryptococcosis (234.47 ± 196.33 vs. 176.05 ± 102.24 *μ*mol·L^−1^, *p*=0.022).

### 3.2. Organ Involvement and Clinical Manifestations

The most commonly affected organs were central nervous system (CNS) (26 patients, 70.3%) and lungs (18 patients, 48.6%). Other involved sites included skin and soft tissue (2 patients, 5.4%) as part of disseminated infection. Disseminated cryptococcosis was identified among 17 patients (45.9%), 10 (27.0%) of which had cryptococcemia and 7 (18.9%) had at least two organ systems involved (4 cases involved CM and lung; 1 case involved CM, lung, and skin abscess; and 1 case involved CM and skin abscess). Furthermore, 7 (18.9%) patients had simple pulmonary infection.

The clinical manifestations in the patients with cryptococcal meningitis (CM) were as follows: fever (24/26, 92.3%), headache (25/26, 96.2%), vomiting (11/26, 42.3%), altered mental status (10/26, 38.5%), seizures (3/26, 11.5%), visual symptoms (2/26, 7.7%), and auditory symptoms (1/26, 3.8%). More patients with disseminated cryptococcosis presented fever than those without (15/17, 88.2% vs. 13/20, 65.0%), but the difference was nonsignificant (*p*=0.137). Cough and expectoration were the primary clinical manifestations in patients with pulmonary disease. Other manifestations included dyspnea and back pain. However, 12/18 patients with pulmonary involvement (66.7%) had no respiratory symptoms, and pulmonary lesions were revealed only by imaging. Among the 12 patients, 9 patients were diagnosed by routine lung CT scan because of symptoms such as fever and headache. Three patients with simple pulmonary infection and no other symptoms were found to have lung lesions on regular follow-up. The average time from the onset of symptoms or pulmonary lesions to diagnosis was 43.95 ± 12.78 (3–360) days.

### 3.3. Laboratory Findings

The average white blood cell (WBC) count was 7,430 ± 3,050 (2,300–14,300) cells per mm^3^. The average C-reactive protein (CRP) level was 26.41 ± 28.00 (0.7–108) mg·L^−1^ (normal range, <8 mg·L^−1^), and the CRP level was elevated in 21 patients (56.8%). Patients with disseminated cryptococcosis had higher WBC counts and elevated CRP levels than those without, but the difference was nonsignificant. Blood culture was positive in 10 patients (27.0%). Other positive culture sites included lung tissue (2 patients), alveolar lavage fluid (1 patient), and soft tissue abscess (2 patients).

Lumbar punctures were performed at baseline in 26 patients with CM. Prior to the administration of antifungal treatment, the average CSF opening pressure was 281.91 ± 25.93 (50–450) mmH_2_O and 42.3% patients (11/26) had an elevated CSF pressure of >300 mmH_2_O. Furthermore, the average WBC count in CSF was 83.93 ± 16.93/mm^3^ (0–310/mm^3^). The average protein concentration in CSF was 0.97 ± 0.10 (0.01–2.27) g·L^−1^, and 92.3% of the patients (24/26) had an average protein concentration of >0.45 g·L^−1^. The average CSF glucose level was 2.25 ± 0.23 (0.10–5.0) mmol·L^−1^ (34.6% [9/26] < 2.0 mmol·L^−1^). CSF cultures were positive for *Cryptococcus* in 18/26 patients (69.2%), whereas India ink smears were positive in 19/26 patients (73.1%). Seven patients (26.9%) were smear negative but culture positive, and 6 patients (23.1%) were culture negative but smear positive. Histologic examinations revealed cryptococcosis in the lungs of 4 patients and the skin of 1 patient.

Only seven patients were tested for serum cryptococcal antigen. Among them, six patients were found to be positive, comprising 2 patients with disseminated cryptococcosis and 4 with cryptococcal pneumonia. The remaining patient tested negative for the antigen and was diagnosed with simple cryptococcal pneumonia by lung biopsy.

### 3.4. Imaging Findings

MR brain images were available for 24 patients with CM, and 16 patients (66.7%) had abnormal results. MRI detected hydrocephalus in 1 patient (4.2%) and local lesions in 16 (66.7%). Local lesions were characterized by a low signal on T1-weighted images and high signal on T2-weighted images and FLAIR in MRI with or without enhancement. The common sites involved included periventricular region (*n* = 9), centrum semiovale (*n* = 7), frontal lobe (*n* = 7), parietal lobe (*n* = 4), basal ganglion (*n* = 3), temporal lobe (*n* = 1), occipitallobe (*n* = 1), and corpus callosum (*n* = 1).

Chest CT images were available for 18 patients with pulmonary disease. The chest CT findings included nodules in 10 patients (55.6%), masses in 6 (33.3%), cavitations in 9 (50%), patchy shadows in 3 (16.7%), pleural effusion in 3 (16.7%), and miliary lesions in 1 with disseminated cryptococcosis (5.5%).

**Figure 1:**
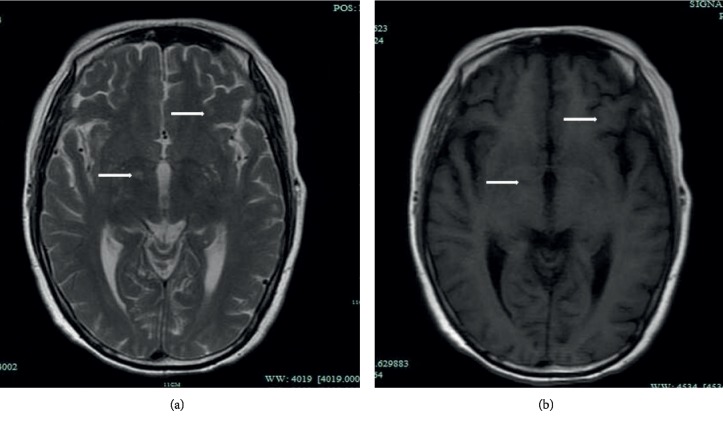
A 53-year-old woman with cryptococcal meningitis with a history of KT 12 years ago. Brain MRI shows local lesions in the right basal ganglion and frontal lobe. (a) Axial T2-weighted image showing a high signal. (b) Axial T1-weighted image showing a low signal.

**Figure 2:**
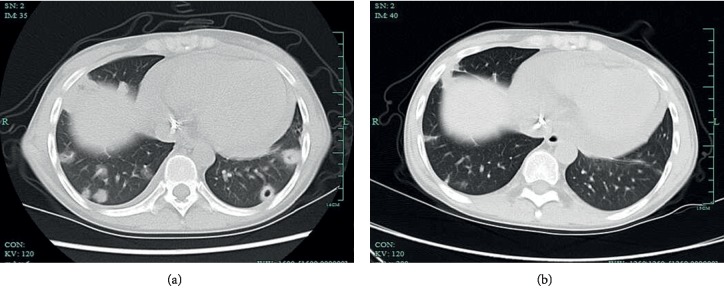
A 20-year-old woman with a history of KT 4 years ago. (a) Chest CT in the lung window shows subpleural multiple nodules, some with cavitations, located in bilateral lungs. (b) Follow-up chest CT (2 months after antifungal treatment) shows the resolution of the pulmonary lesions.

The typical abnormalities on cranial MRI and chest CT are shown in Figures 1 and 2.

### 3.5. Treatment and Outcomes

In our study, the 2-week mortality rate was 8.1% (3/37), and 5/37 patients (13.5%) died within 6 months of being diagnosed with cryptococcosis. Three patients died within 2 weeks, two of which were diagnosed with CM and one was diagnosed with disseminated cryptococcosis. All deaths within 2 weeks were attributed to cryptococcosis. Among the five patients who died within 6 months, three were diagnosed with CM and two were diagnosed with disseminated cryptococcosis. One patient with disseminated cryptococcosis who received liposomal-AMB therapy died after 4 months of initial therapy because of severe septicemia. One patient with CM who received liposomal-AMB died after 7 months of initial therapy because of severe infective endocarditis. However, one patient who received 400 mg of FLZ daily for induction and maintenance therapy died of cryptococcemia 10 months after the initiation of antifungal treatment. All patients with simple pulmonary infections survived during follow-up.

The initial treatments were classified as follows: 7 patients, liposomal-AMB combined with 5-FC; 22, FLZ ± 5-FC; 7, VRZ ± 5-FC; and 1, itraconazole alone. 7 patients with CM and/or disseminated cryptococcosis in our study received domestic liposomal-AMB plus 5-FC for initial treatment. One patient who received 120 mg dosage of liposomal-AMB (about 2.0 mg/kg) daily for initial treatment had elevated serum creatinine three times higher than baseline after 7 days, and it returned to normal after we changed to FLZ 400 mg. The other 6 patients received liposomal-AMB 40 to 70 mg per day (about 0.8–1.0 mg/kg per day) for initial treatment. The average duration of liposomal-AMB treatment was 20.00 ± 4.57 (7–42) days in the 7 patients. As shown in Table 2, the all-cause 6-month mortality rate in patients with CM or disseminated cryptococcosis was 28.6% (2/7) in the liposomal-AMB combined with 5-FC group, 17.6% (3/17) in the FLZ ± 5-FC group, and 0% in the VRZ ± 5-FC and itraconazole groups. The mortality rate of patients with CM or disseminated cryptococcosis who received triazole (13.0%, 3/23) was lower than that of patients treated with liposomal-AMB (28.6%, 2/7). However, the difference was nonsignificant (*p*=0.565). Further analysis revealed that all patients in the high-dose FLZ (800 mg daily) and VRZ ± 5-FC groups survived. All patients received FLZ or VRZ for consolidation and maintenance therapy.

Graft loss was observed in 3 patients prior to antifungal treatment, with 1 having received liposomal-AMB + 5-FC and 2 having received FLZ + 5-FC. Chronic rejection led to graft loss in these three patients. Five additional patients, who had preexisting renal dysfunction (including one treated with liposomal-AMB, two with FLZ, and two with VRZ for induction therapy), progressed to graft loss after starting antifungal treatment. No significant difference was found in terms of graft loss between patients who received liposomal-AMB and those who received triazole (16.7% vs. 14.3%, *p*=0.881).

## 4. Discussion

We performed the first allograft KT in July 1977 at the First Affiliated Hospital of School of Medicine, Zhejiang University, which is the main transplantation centre in China. However, opportunistic infections, including invasive fungal or viral infections and tuberculosis, remain a concern in patients who have undergone KT in terms of successful long-term outcomes [1, 13]. Only a small number of cases of post-KT cryptococcosis have been reported in mainland China [9–11]. In the present study, 37 patients diagnosed with post-KT cryptococcosis and their characteristics were analyzed.

Our findings showed that cryptococcosis in KT recipients is typically a late-occurring infection. Approximately 81.1% patients (30/37) had cryptococcosis onset after >2 years following KT. These findings are in agreement with those of previous reports [1, 5, 9, 14]. The results of a multicenter prospective surveillance from the United States [1] showed that the median time for cryptococcosis onset is 575 days following KT, and 75% of the cases occurred >3 years following KT. Another large retrospective cohort study from the United States showed the median time of cryptococcosis onset is 616 days following KT [14], similar to the findings of a recent overview of cryptococcosis following KT in China [9], which revealed that 93.1% of the infections occurred >1 year following transplantation and 79.3% occurred >3 years following transplantation. According to the literature, the majority of cryptococcosis onset in KT recipients is late and is generally considered to represent the reactivation of a latent infection [1, 14]; however, early-onset infections may reflect donor-derived infections [15, 16].

Previous studies [4, 9, 17, 18] have shown that the majority of cryptococcal infections in KT recipients involve the CNS, lungs, skin, and soft tissue. The present study also revealed that the most common site is the CNS, followed by the lungs, skin, and soft tissue. Similar to the findings of a previous study [9], fever, headache, vomiting, and altered mental status were found to be the most common clinical manifestations of post-KT CM.

According to the IDSA guidelines from 2010 [12], induction therapy with the liposomal-AMB or AMB lipid complex combined with 5-FC is recognised as the preferred regimen for CM, disseminated cryptococcosis, and severe pulmonary cryptococcosis in SOT recipients. However, in the present study, apparent differences exist between the regime we adopted and those recommended in the guidelines. 7 simple cryptococcal pneumonia patients received triazole treatment. Among the 30 patients with CM or disseminated cryptococcosis, only 7 patient (23.3%, 7/30; 4 with CM and 3 with disseminated cryptococcosis) were treated with liposomal-AMB combined with 5-FC during the induction phase. A similarly low use of liposomal-AMB was reported in other Chinese centres [9] which we attribute to the fear of side effects. It is possible that the renal toxicity side effects associated with liposomal-AMB resulted in its decreased use in our population. In the present study, graft dysfunction was common in KT recipients with cryptococcosis prior to treatment initiation, and the baseline creatinine level was >176.8 *μ*mol·L^−1^ in 37.8% of the patients. However, no significant difference in terms of graft loss was found between the patients who received liposomal-AMB and triazole (16.7% vs. 14.3%, *p*=0.88).

In the present study, the overall prognosis of cryptococcosis following KT was good. All cases of simple pulmonary infection survived following the administration of triazole treatment. Among patients with CM or disseminated cryptococcosis, the all-cause 6-month mortality rate in those who received triazole (13.0%, 3/23) was lower than that in patients who received liposomal-AMB (28.6%, 2/7); however, the difference was not statistically significant (*p*=0.565). The study also showed that all patients who received high-dose FLZ (800 mg daily) or VRZ ± 5-FC as the induction treatment survived.

FLZ is widely used for the treatment of fungal diseases due to its effectiveness, low cost, and availability in oral and intravenous preparations. The IDSA guidelines recommend high-dose FLZ (≥800 mg daily), either alone or with other antifungal drugs, as an alternative anticryptococcal choice. It is also recommended for CM as consolidation and maintenance therapy and for mild-to-moderate pulmonary cryptococcosis [12]. VRZ is a triazole with a broad spectrum of antifungal activity but is not currently licensed for use for cryptococcosis. Case studies have demonstrated the successful treatment of CM with VRZ [19–22]. In vitro activities of VRZ [23, 24] show its better activity against *Cryptococcus neoformans* than itraconazole and FLZ, suggesting that VRZ is useful in the treatment of cryptococcosis.

Our study suggested that high-dose FLZ or VRZ alone or with 5-FC may be used as an alternative therapy for post-KT cryptococcosis. However, further investigations on optimizing antifungal treatment and preventing the interaction of triazole with immunosuppressive agents among KT recipients in China should be carried out in the future.

The present study has several limitations. The study was designed as a retrospective study performed at a single tertiary hospital. Furthermore, it reflected a single-centre experience and the number of enrolled patients was relatively small, which may have led to several biases. Therefore, whether our findings are representative of the entire Chinese population is uncertain. Further large multicentre and prospective studies are required to obtain accurate data regarding cryptococcosis in KT recipients in the Chinese population.

In conclusion, most patients with post-KT cryptococcosis experienced cryptococcosis onset >2 years following KT at our hospital. The CNS, pulmonary system, and skin are the main sites of infection. Triazole can be used as an alternative therapy for post-KT cryptococcosis.

## Figures and Tables

**Table 1:** Demographic and clinical data of KT recipients with cryptococcosis (*n* = 37).

Variable	*N* (%)
Male sex	23 (62.3%)

Age at diagnosis (years)	49.5 ± 9.38

Underlying diseases	
Hypertension	15 (40.5%)
Chronic viral hepatitis B	10 (27.0%)
Type 2 diabetes	5 (13.5%)
Chronic viral hepatitis C	3 (8.1%)
Systemic lupus erythematosus	1 (2.7%)

Immunosuppressive regimen	
Prednisone	37 (100%)
Mycophenolate mofetil	30 (80.1%)
Mycophenolic acid	1 (2.7%)
Tacrolimus	29 (78.4%)
Cyclosporine A	8 (21.6%)
Azathioprine	2 (5.4%)
Leflunomide	2 (5.4%)
Rapamycin	2 (5.4%)

Posttransplantation time to diagnosis of cryptococcosis (years)	7.0 ± 5.50
<2 year	7 (18.9%)
>2 years	30 (81.1%)

Organ involvement	
Central nervous system	26 (70.3%)
Lungs	18 (48.6%)
Skin	2 (5.4%)

Disseminated cryptococcosis	17 (45.9%)

Cryptococcemia	10 (27.0%)

Baseline creatinine level (*μ*mol·L^−1^)	202.90 ± 153.40

**Table 2:** Six-month outcome of cryptococcosis with different initial antifungal therapies.

Initial therapy	Total				CM or disseminated cryptococcosis			Lung disease only		
	Total	Evaluated	Death	Mortality (%)	Evaluated	Death	Mortality (%)	Evaluated	Death	Mortality
Liposomal-AMB + 5-FC	7	7	2	28.6	7	2	28.6	0	0	0
FLZ ± 5-FC	22	22	3	13.6	17	3	17.6	5	0	0
FLZ 400 mg daily ± 5-FC	18	18	3	16.7	14	3	21.3	4	0	0
FLZ 800 mg daily ± 5-FC	4	4	0	0	3	0	0	1	0	0
VRZ ± 5-FC	7	7	0	0	5	0	0	2	0	0
Itraconazole	1	1	0	0	1	0	0	0	0	0

## Data Availability

The data used to support the findings of this study are included within the article.

## References

[1] Pappas P. G., Alexander B. D., Andes D. R. (2010). Invasive fungal infections among organ transplant recipients: results of the transplant-associated infection surveillance network (TRANSNET). *Clinical Infectious Diseases*.

[2] Gassiep I., McDougall D., Douglas J., Francis R., Playford E. G. (2017). Cryptococcal infections in solid organ transplant recipients over a 15-year period at a state transplant center. *Transplant Infectious Disease*.

[3] Chen M., Xu Y., Hong N. (2018). Epidemiology of fungal infections in China. *Frontiers of Medicine*.

[4] Sun H. Y., Wagener M. M., Singh N. (2009). Cryptococcosis in solid-organ, hematopoietic stem cell, and tissue transplant recipients: evidence-based evolving trends. *Clinical Infectious Diseases*.

[5] Neofytos D., Fishman J. A., Horn D. (2010). Epidemiology and outcome of invasive fungal infections in solid organ transplant recipients. *Transplant Infectious Disease*.

[6] Singh N., Dromer F., Perfect J. R., Lortholary O. (2008). Immunocompromised hosts: cryptococcosis in solid organ transplant recipients: current state of the science. *Clinical Infectious Diseases*.

[7] Shi B. Y. L. Z. (2018). Kidney transplantation: from quality control to quality improvement plan. *Chinese Journal of Transplantation*.

[8] Yuchong C., Fubin C., Jianghan C. (2012). Cryptococcosis in China (1985–2010): review of cases from Chinese database. *Mycopathologia*.

[9] Yang Y.-L., Chen M., Gu J.-L. (2014). Cryptococcosis in kidney transplant recipients in a Chinese university hospital and a review of published cases. *International Journal of Infectious Diseases*.

[10] Chen M., Wang X., Yu X. (2015). Pleural effusion as the initial clinical presentation in disseminated cryptococcosis and fungaemia: an unusual manifestation and a literature review. *BMC Infectious Diseases*.

[11] Sang H., Zhou W. Q., Shi Q. L., Zhang X. H., Ni R. Z. (2004). Disseminated cryptococcosis with extensive subcutaneous nodules in a renal transplant recipient. *Chinese Medical Journal*.

[12] Perfect J. R., Dismukes W. E., Dromer F. (2010). Clinical practice guidelines for the management of cryptococcal disease: 2010 update by the infectious diseases society of America. *Clinical Infectious Diseases*.

[13] Wu W., Yang M., Xu M. (2018). Diagnostic delay and mortality of active tuberculosis in patients after kidney transplantation in a tertiary care hospital in China. *PLoS One*.

[14] George I., Santos C., Olsen M. A., Powderly W. G. (2017). Epidemiology of cryptococcosis and cryptococcal meningitis in a large retrospective cohort of patients after solid organ transplantation. *Open Forum Infectious Diseases*.

[15] Camargo J. F., Simkins J., Schain D. C. (2018). A cluster of donor-derived *Cryptococcus neoformans* infection affecting lung, liver, and kidney transplant recipients: case report and review of literature. *Transplant Infectious Disease*.

[16] Baddley J. W., Schain D. C., Gupte A. A. (2011). Transmission of *Cryptococcus neoformans* by organ transplantation. *Clinical Infectious Diseases*.

[17] Husain S., Wagener M. M., Singh N. (2001). *Cryptococcus neoformans* infection in organ transplant recipients: variables influencing clinical characteristics and outcome. *Emerging Infectious Diseases*.

[18] Ponzio V., Camargo L. F., Medina-Pestana J., Perfect J. R., Colombo A. L. (2018). Outcomes of cryptococcosis in renal transplant recipients in a less-resourced health care system. *Transplant Infectious Disease*.

[19] Li S.-S., Tang X.-Y., Zhang S.-G., Ni S.-L., Yang N.-B., Lu M.-Q. (2016). Voriconazole combined with low-dose amphotericin B liposome for treatment of cryptococcal meningitis. *Infectious Diseases*.

[20] Yao Y., Zhang J.-T., Yan B. (2015). Voriconazole: a novel treatment option for cryptococcal meningitis. *Infectious Diseases*.

[21] Bandettini R., Castagnola E., Calvillo M. (2009). Voriconazole for cryptococcal meningitis in children with leukemia or receiving allogeneic hemopoietic stem cell transplant. *Journal of Chemotherapy*.

[22] Sabbatani S., Manfredi R., Pavoni M., Consales A., Chiodo F. (2004). Voriconazole proves effective in long-term treatment of a cerebral cryptococcoma in a chronic nephropathic HIV-negative patient, after fluconazole failure. *Mycopathologia*.

[23] Pfaller M. A., Zhang J., Messer S. A. (1999). In vitro activities of voriconazole, fluconazole, and itraconazole against 566 clinical isolates of *Cryptococcus neoformans* from the United States and Africa. *Antimicrobial Agents and Chemotherapy*.

[24] Xiao M., Chen S. C. A., Kong F. (2018). Five-year China Hospital Invasive Fungal Surveillance Net (CHIF-NET) study of invasive fungal infections caused by noncandidal yeasts: species distribution and azole susceptibility. *Infection and Drug Resistance*.

